# Revisiting vitamin D status and supplementation for inpatients with intellectual and developmental disability in the north of England, UK

**DOI:** 10.1192/bjo.2021.95

**Published:** 2021-06-18

**Authors:** Vlad Ciausu, Marcin Ostrowski, Bethany Dudley, Iain McKinnon, Chris Ince

**Affiliations:** 1 Cumbria, Northumberland, Tyne And Wear NHS Foundation Trust; 2 Newcastle University, Cumbria, Northumberland, Tyne And Wear NHS Foundation Trust

## Abstract

**Aims:**

Vitamin D deficiency is common among people with Intellectual and Developmental Disability (IDD) and is linked to worse health outcomes.

Our aims were to re-evaluate vitamin D testing and supplementation among inpatients with IDD, examine any correlates with physical health conditions including COVID-19 and make recommendations for the current regime of supplementation and testing within inpatient IDD services.

**Method:**

The study population comprised inpatients who were in any of the Northgate Hospital IDD inpatient services in Northumberland, UK. The wards sampled were the Medium Secure Unit, Low Secure Unit, Hospital Based Rehabilitation Wards and Specialist Autism Inpatient Service. Records of all inpatients between January 2019 and July 2020 were examined for 25-hydroxyvitamin D [25(OH)D] level, ward area, supplementation status, test seasonality, medication, and health status.

We performed a correlation to see whether there was an association between vitamin D level and length of time on treatment. In addition, comparison of the replete and inadequate group for age, ethnicity, seasonality, ward location and psychotropic medication was undertaken.

Data on physical health risk factors, obesity and COVID-19 infection were also collected. The physical comorbidities were described in order to evaluate whether any emerging patterns relating to COVID-19 infection were emerging.

**Result:**

There were 67 inpatients in Northgate IDD services on 1 January 2019, with 11 further patients admitted up to the end of the sampling period on 31 July 2020. Nineteen patients were discharged during that period, so the sample comprised 78 patients.

Ages were comparable across three of the ward areas, except for an older group of patients in the hospital-based rehabilitation setting. Mean 25(OH)D level for supplemented (800IU/day) patients was 75nmol/l (SD 20) compared to 40nmol/l (SD 19) in the non-supplemented group (p < 0.001).

Thirty-eight percent of those who were inpatients during the first wave of the COVID-19 pandemic developed symptoms, but the small sample size could not establish vitamin D levels as a predictor of outcome.

**Conclusion:**

Our findings show that clinicians continue to offer vitamin D supplementation for inpatients, at a dose of 800IU (20μg) per day.

The mean vitamin D levels we observed were higher for those on supplements compared to our 2013 baseline data, whereas patients not on supplementation now had levels akin to those found previously. Vitamin D (800IU/day) supplementation is effective but adequacy of the nationally recommended dose of 400IU/day is unclear. Links to COVID-19 merit further research.

